# Systematic review and meta-analysis of the management of acute uncomplicated diverticulitis: time to change traditional practice

**DOI:** 10.1007/s00384-024-04618-7

**Published:** 2024-04-05

**Authors:** Ali Yasen Mohamedahmed, Shafquat Zaman, Niloy Das, Georgios Kakaniaris, Stelios Vakis, James Eccersley, Pradeep Thomas, Najam Husain

**Affiliations:** 1 Department of General and Colorectal Surgery, Queen’s Hospital Burton, University Hospital of Derby and Burton NHS Trust, Derby, UK; 2 Department of General Surgery, Russells Hall Hospital, The Dudley Group NHS Foundation Trust, Dudley, West Midlands, UK; 3 Institute of Cancer and Genomic Science, College of Medical and Dental Science, University of Birmingham, Edgbaston, Birmingham, UK

**Keywords:** Acute diverticulitis, Hinchey 1a, Systematic review

## Abstract

**Background:**

To evaluate comparative outcomes of outpatient (OP) versus inpatient (IP) treatment and antibiotics (ABX) versus no antibiotics (NABX) approach in the treatment of uncomplicated (Hinchey grade 1a) acute diverticulitis.

**Methods:**

A systematic online search was conducted using electronic databases. Comparative studies of OP versus IP treatment and ABX versus NABX approach in the treatment of Hinchey grade 1a acute diverticulitis were included. Primary outcome was recurrence of diverticulitis. Emergency and elective surgical resections, development of complicated diverticulitis, mortality rate, and length of hospital stay were the other evaluated secondary outcome parameters.

**Results:**

The literature search identified twelve studies (*n* = 3,875) comparing NABX (*n* = 2,008) versus ABX (*n* = 1,867). The NABX group showed a lower disease recurrence rate and shorter length of hospital stay compared with the ABX group (*P* = 0.01) and (*P* = 0.004). No significant difference was observed in emergency resections (*P* = 0.33), elective resections (*P* = 0.73), development of complicated diverticulitis (*P* = 0.65), hospital re-admissions (*P* = 0.65) and 30-day mortality rate (*P* = 0.91). Twelve studies (*n* = 2,286) compared OP (*n* = 1,021) versus IP (*n* = 1,265) management of uncomplicated acute diverticulitis. The two groups were comparable for the following outcomes: treatment failure (*P* = 0.10), emergency surgical resection (*P* = 0.40), elective resection (*P* = 0.30), disease recurrence (*P* = 0.22), and mortality rate (*P* = 0.61).

**Conclusion:**

Observation-only treatment is feasible and safe in selected clinically stable patients with uncomplicated acute diverticulitis (Hinchey 1a classification). It may provide better outcomes including decreased length of hospital stay. Moreover, the OP approach in treating patients with Hinchey 1a acute diverticulitis is comparable to IP management. Future high-quality randomised controlled studies are needed to understand the outcomes of the NABX approach used in an OP setting in managing patients with uncomplicated acute diverticulitis.

**Supplementary Information:**

The online version contains supplementary material available at 10.1007/s00384-024-04618-7.

## Introduction

Acute colonic diverticulitis is a common surgical presentation in the emergency setting [1]. Although the ‘true’ incidence of diverticulosis and diverticular disease remains unknown, global prevalence is increasing in both developed and developing countries and is often linked with dietary and lifestyle modifications [2]. 

Colonic diverticula can occur in any part of the colon but are often localised to the descending and sigmoid segments. The exact aetiology for the development of these sac-like protrusions remains unclear, but a number of changes in the wall of the colon, including loss of elasticity function, are known to occur [2]. Neuromuscular abnormalities with changes in the enteric nervous system and collagen deposition in the presence of increased intraluminal pressure are thought to be underlying mechanisms.

Acute diverticulitis ranges in severity from a mild, self-limiting illness (peri diverticular inflammation limited to the colonic wall) to a complicated disease characterised by sepsis, abscess formation, haemorrhage, and perforation necessitating urgent surgical intervention. Pro-inflammatory biomarkers such as C-reactive protein (CRP) levels remain the most useful predictors of disease severity [3]. 

Multiple clinical and radiological scoring systems are available for grading diverticulitis [4]. The most widely used is the Hinchey classification [5], which has since undergone several modifications following the introduction of computed tomography (CT) [6–8]. These modifications include additional subcategories considering radiological findings and range from mild clinical disease (stage 0) to generalised faecal peritonitis (grade IV) [7]. Consequently, therapeutic options are broad, including medical management (analgesia, probiotics, dietary fibre, antibacterial), radiological (percutaneous interventions), and elective/emergency surgery.

Consensus on optimal treatment is lacking, and the long-standing recommendation of systemic antibiotics (ABX) to routinely treat acute diverticulitis has recently been challenged [9]. Current guidelines suggest the adoption of a no-antibiotic (NABX) strategy in treating patients with acute diverticulitis without systemic upset (uncomplicated cases) [10]. Moreover, in selected patients (immunocompetent, tolerating oral intake, low CRP, absence of fever) with uncomplicated diverticulitis, in addition to omission of antibacterials, outpatient (OP) management may also be safe and feasible [1]. 

We performed a systematic review and meta-analysis of the available literature to assess outcomes comparing ABX vs. NABX and inpatient (IP) vs. OP management in patients presenting with Hinchey Ia disease (defined as confined pericolic inflammation or phlegmon).

## Methods

This systematic review was designed, performed, and reported as per the recommendations of the Cochrane Handbook for Systematic Reviews of Interventions and Preferred Reporting Items for Systematic Reviews and Meta-Analyses (PRISMA) guidelines [11, 12]. The protocol of this review was registered in PROSPERO (ID: CRD 42023488826).

Studies included in this analysis for the comparison of ABX versus NABX treatment were based on the following PICO (Population, Intervention, Comparator, Outcomes):

- **P:** Patients presenting with primary or recurrent uncomplicated diverticulitis (defined as Hinchey stage 1a) diagnosed radiologically via a CT scan.
- **I:** Observational treatment without the use of antibacterial therapy.
- **C:** Treatment with intravenous or oral antibiotics.
- **O:** Recurrence of diverticulitis during the maximum follow-up period, emergency surgical resection, elective surgical resection, development of complicated diverticulitis, mortality rate, and length of hospital stay.

### Study design

This study was conducted as a systematic review and meta-analysis of comparative studies. Single-arm studies, case series / case reports, and letters to the editor were excluded.

For comparison between OP and IP treatment of patients presenting with acute uncomplicated diverticulitis, the following PICO was used:

- **P:** Patients presenting with primary or recurrent uncomplicated diverticulitis (defined as Hinchey stage 1a) diagnosed radiologically via a CT scan.
- **I:** Outpatient or ambulatory treatment.
- **C:** Inpatient treatment (defined as admission to hospital).
- **O:** Treatment failure, emergency and elective surgical resection, recurrence of diverticulitis and mortality rate.

### Study design

This study was conducted as a systematic review and meta-analysis of comparative studies. Single-arm studies, case series/case reports, and letters to the editor were excluded.

### Search strategy

Comparative studies comparing OP versus IP treatment or ABX versus NABX for Hinchey 1a acute diverticulitis were deemed eligible for inclusion. The literature search was performed using PubMed, MEDLINE, Embase and Cochrane Central Register of Controlled Trials (CENTRAL) up to and including 1 December 2023 with no language restrictions.

Moreover, the reference list of the relevant studies was reviewed manually for potential eligible studies. A combination of the following search terms was used: “uncomplicated acute diverticulitis”, “Hinchey 1a diverticulitis”, “ambulatory”, “outpatient”, “inpatient” and “uncomplicated acute diverticulitis”, “Hinchey 1a diverticulitis”, “no antibiotics”, “without antibiotics”, “observation”, “antibiotics”, “anti-microbial”, “anti-bacterial” to retrieve studies comparing outpatient versus inpatient treatment and antibiotic versus no-antibiotic treatment, respectively. Search strategy is outlined in Appendix 1.

Two authors independently searched the previously mentioned electronic databases, and two authors reviewed the extracted studies/data.

### Eligibility and study selection criteria

Studies comparing OP versus IP management or ABX versus NABX treatment for Hinchey 1a acute diverticulitis were included. Studies comparing patients presenting with complicated grades of acute diverticulitis (defined as Hinchey stage 1b, 2, 3 and 4) were excluded. Moreover, non-comparative (single-arm) studies, case series, and case reports were also excluded.

Titles and abstracts of selected articles were screened independently by two authors, and the full text of potentially eligible articles was retrieved. Disagreements were resolved through consensus or consultation with the senior author.

### Data extraction and outcomes

Two authors extracted data independently and revised by a third author using an Excel spreadsheet. The information collected from each study included the name of the author, year of publication, study design, total number of patients, inclusion and exclusion criteria, follow-up period, patient demographics, and relevant outcomes.

In the first comparison (ABX versus NABX), the primary outcome was disease recurrence. Other measured metrics (secondary outcomes) were emergency resection rate, elective resections, development of complicated diverticulitis, mortality rate, and length of hospital stay.

Treatment failure was considered the primary outcome for our secondary comparison (OP versus IP management). Treatment failure was defined as the need for hospital admission directly related to or as a consequence of index pathology. Secondary outcomes were emergency surgical resection, elective resection, recurrence of diverticulitis, and mortality rate.

### Risk of bias assessment

The Cochrane risk of bias tool and the Newcastle-Ottawa Scale (NOS) were used to assess the risk of bias in the included RCTs and observational studies [13, 14]. Studies were considered low, medium, or high risk of bias if the total NOS score was 9, 7/8, or less than 6, respectively. Disagreements during this process were resolved through discussion and consultation with the authorship team.

### Data synthesis and statistical analyses

The meta-analysis was performed using RevMan version 5.3. Dichotomous outcomes were pooled with a random-effects model to estimate the odds ratio (OR) or risk difference (RD) (where more than three studies reported zero events in both groups) with a 95% confidence interval (CI).

A mean difference (MD) with 95% CI was estimated for continuous outcomes. The Hozo et al. [15] equation was used to estimate mean and standard deviation (SD) when continuous variables were reported as median and interquartile range (IQR).

The results were considered statistically significant if the *P*-value was < 0.05 or if the 95% CI did not include 1. Heterogeneity was evaluated using the Cochran Q test (χ2) and I^2^ statistic. An I^2^ value exceeding 50% signified significant levels of heterogeneity, whilst a value of 0% indicated no heterogeneity.

To check for possible sources of heterogeneity and evaluate the robustness of the results, sensitivity analysis was performed by calculating the risk ratio (RR) or RD for dichotomous variables. Moreover, a ‘leave-one-out’ analysis was conducted to assess each study’s effect individually.

## Results

### A- Antibiotics versus no antibiotics

**Fig. 1:**
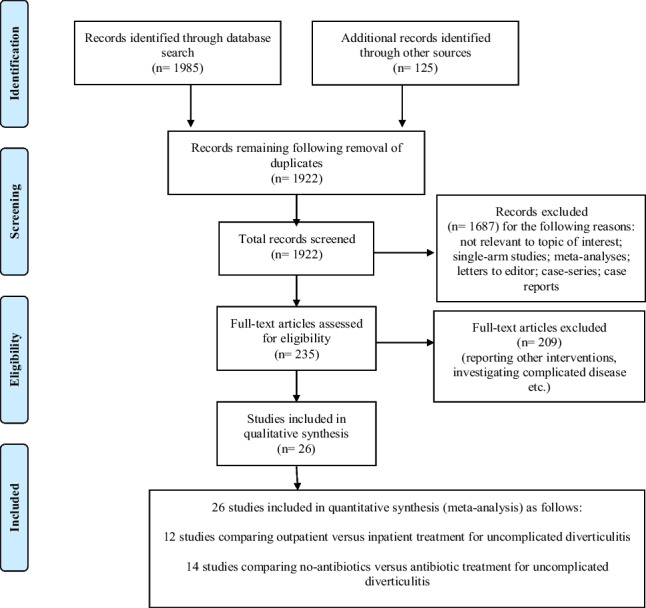
PRISMA flow chart

Our search yielded twelve studies [16–29] comparing NABX versus ABX treatment in patients diagnosed with Hinchey 1a diverticulitis (PRISMA flow chart - Fig. 1). The total number of patients (*n* = 3,875) was divided between the NABX group (*n* = 2,008) and the ABX group (*n* = 1,867). Five of the included studies were multicentric RCTs [17, 18, 21, 22, 24, 25, 27], and two of the included trials (AVOD & DIABOLO) have been reported in two papers each. Two of the studies [24, 26] included right-side colonic diverticulitis exclusively, and three [21, 22, 26] included patients presenting with a first episode of acute diverticulitis. Characteristics of the included studies are summarised in Table 1.

**Table 1:** Baseline characteristics of included studies comparing no-antibiotics versus antibiotic treatment for patients with uncomplicated diverticulitis

**Study**	**Country**	**Study Type**	**Number** **of patients**	**Inclusion and Exclusion criteria**	**Hinchey stage and follow-up duration (months)** **mean ± SD/** **median(range)**
Hjern et al. [16]	Sweden	Retrospective Cohort	NABX: 193 ABX: 118	**Exclusion criteria**: diagnosis based on clinical findings only without CT scan, perforated AD on CT scan	**Hinchey Stage** 1a 30 months
AVOD trial (Chabok et al. [17] and Isacson et al. [18])	Sweden & Iceland	Multicentric RCT	NABX: 309 ABX: 314	**Inclusion criteria**: adult patients > 18 years, acute lower abdominal pain with tenderness, signs of AD on CT, informed consent **Exclusion criteria**: complicated AD on CT with abscess, fistula or free air in abdomen or pelvis, other diagnoses on CT, immunosuppressive therapy, pregnancy, ongoing antibiotic therapy, high fever, peritonitis/sepsis	**Hinchey Stage** 1a NABX: 132 (13–173) ABX: 132 (8-165)
de Korte et al. [19]	Netherlands	Multicentric Case-Control	NABX: 191 ABX: 81	**Inclusion criteria**: imaging-confirmed (CT scan) acute mild (Ambrosetti) or Hinchey 1a AD of the sigmoid colon	**Hinchey Stage** 1a 50 months (12–100)
Brochmann et al. [20]	Norway	Retrospective Cohort	NABX: 174 ABX: 46	**Inclusion criteria**: CT-verified, left-sided, colonic acute uncomplicated AD **Exclusion criteria**: ongoing antibiotic treatment at admission, pregnancy, clinical signs of severe illness: body temperature > 39.5 °C, peritonitis, sepsis, severely compromised general condition, and immunocompromised patients	**Uncomplicated AD** 12 months
DIABOLO trial (Daniels et al. [21] and van Dijk et al. [22])	Netherlands	Multicentric RCT	NABX: 262 ABX: 260	**Inclusion criteria**: patients with the first left-sided, uncomplicated AD episode confirmed within 24 h by CT. Only modified Hinchey stages 1a–b **Exclusion criteria**: previous radiologically proven AD, higher modified Hinchey stages or Ambrosetti’s ‘severe’ diverticulitis stage plus sepsis	**Hinchey Stages** 1a–b 24 months
Estrada Ferrer et al. [23]	Spain	Prospective Cohort	NABX: 45 ABX: 32	**Inclusion criteria**: age 18–80 years, no AD episode in the last 3 months, mild AD on CT scan, immunocompetence (no corticosteroid therapy). No significant comorbidities (diabetes mellitus, renal insufficiency, morbid obesity). Good oral tolerance and good symptom control by oral medication.	**Mild AD (mNeff 0)** 6 months (3–12)
Kim et al. [24]	South Korea	RCT	NABX: 66 ABX: 66	**Inclusion criteria**: age 18–80 years, right-sided uncomplicated AD (grade Ia) **Exclusion criteria**: sepsis, systemic inflammatory response syndrome (SIRS), immunocompromised patients, allergy to quinolone antibiotics, pregnant or lactating patients, ASA score > 3, social psychiatric, or cognitive impairment	**Hinchey Stage** 1a NABX 14.7 months ABX 13.5 months
DINAMO trial (Mora-Lopez et al. [25])	Spain	Multicentric RCT	NABX: 242 ABX: 238	**Inclusion criteria**: age between 18–80 years, modified Neff 0 AD on abdominal CT scan, no AD episode in the last 3 months **Exclusion criteria**: pregnancy or breastfeeding, allergy to any of the study drugs, inflammatory bowel disease, antibiotic treatment for any reason in the last 2 weeks, significant comorbidities or immunodepression.	**Hinchey Stage** 1a 3 months
Lee et al. [26]	South Korea	Propensity Score-Matched	NABX: 55 ABX: 55	**Inclusion criteria**: uncomplicated primary right colonic AD. **Exclusion criteria**: recurrent AD, complicated AD, refusal of treatment and subsequent discharge from the hospital, and death during hospitalisation for reasons not related to diverticulitis.	**Hinchey Stage** 1a 229.3 ± 21.9 days
STAND trial (Jaung et al. [27])	New Zealand & Australia	Multicentric RCT	NABX: 94 ABX: 84	**Inclusion criteria**: ≥18 years of age, CT-proven Hinchey AD. **Exclusion criteria**: >2 criteria of SIRS upon presentation, pregnancy, ASA ≥ 4, previous drug reactions to the antibiotics, used any of the following before presentation: steroids, immunomodulators or biologics, regular nonsteroidal anti-inflammatory drugs, or antibiotics.	**Hinchey Stage** 1a 1 month
Azhar et al. [28]	Sweden	Retrospective Cohort	NABX: 195 ABX: 388	**Inclusion criteria**: uncomplicated AD diagnosed on CT (absence of complications such as abscess, fistula, stricture, bowel obstruction, or peritonitis with perforation) **Exclusion criteria**: general peritonitis or sepsis, immunosuppressed patients and patients with ongoing antibiotic treatment at admission	**Uncomplicated AD** 3 months
Serrano Gonzalez et al. [29]	Spain	Prospective Cohort	NABX: 182 ABX: 179	**Inclusion criteria**: uncomplicated AD on CT, age between 18–70 years, ASA I-III, sufficient social and/or family support, patient able to tolerate orally **Exclusion criteria**: complicated AD (evidence of stenosis, abscess, pneumoperitoneum or fistula on CT, or signs of haemorrhage, obstruction or sepsis), pregnancy, immunosuppressed patients, BMI ≥ 40 kg/m2	**Uncomplicated AD** 24 months

* NABX* no antibiotics, *ABX* antibiotics, *AD* acute diverticulitis, *CT* computed tomography, *RCT* randomised controlled trial, *BMI* body mass index, *SD* standard deviation

### Primary outcome

#### Disease recurrence during follow-up

##### All studies

**Fig. 2:**
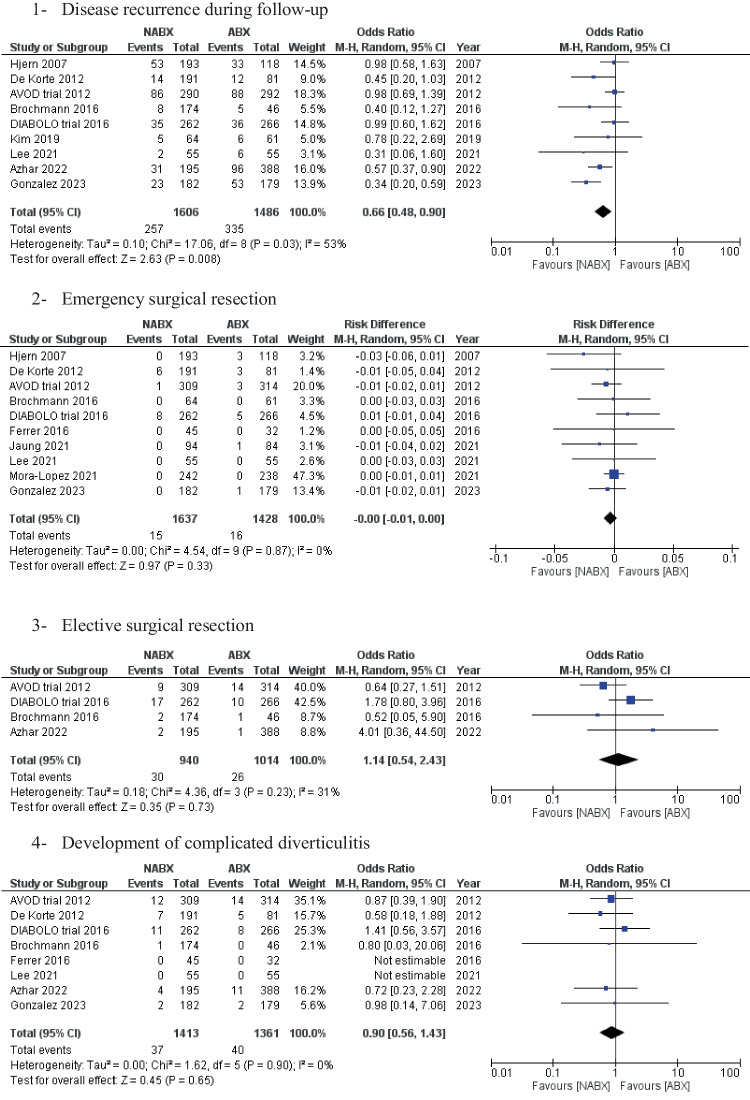

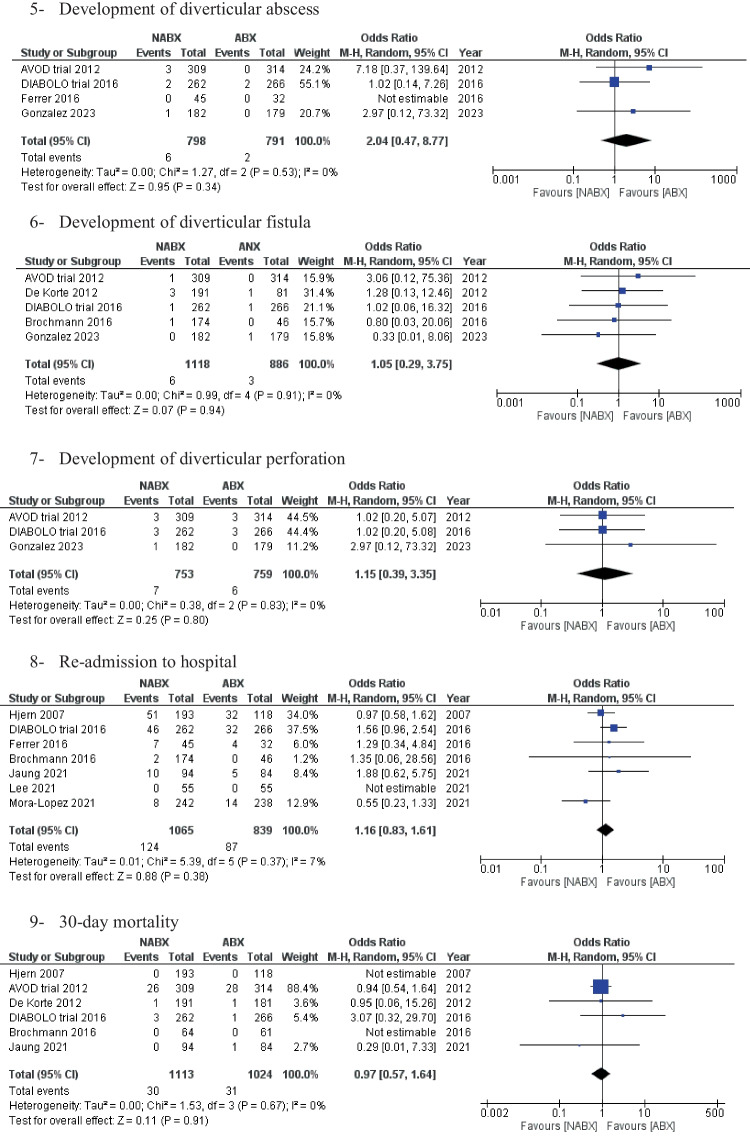

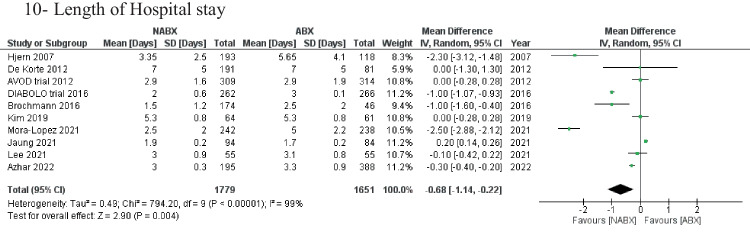
Forest plots of the measured outcomes compared between the no-antibiotic group (NABX) and the antibiotic group (ABX)

Recurrence of diverticulitis during the follow-up period was reported in nine studies, with 3,092 patients (Fig. 2). The total recurrence rate was 19.1% across the two groups. The NABX group was associated with a statistically significantly lower risk of disease recurrence than the ABX group [16% vs. 22.5%, OR: 0.66 (0.84,0.90) 95% CI, *P* = 0.01]. Cochran’s Q test demonstrated substantial heterogeneity amongst the included studies [I^2^ = 53%, *P* = 0.03].

##### Subgroup analysis

Subgroup analysis showed a non-statistically significant trend towards lower recurrence rate in the NABX group as follows: RCTs only [20.4% NABX vs. 21% ABX, OR: 0.97, *P* = 0.82], right-sided diverticulitis [5.8% NABX vs. 10.3% ABX, OR: 0.56 (0.21, 1.50) 95% CI, *P* = 0.25], and first episode of acute diverticulitis [10% NABX vs. 13.4% ABX, OR: 0.64 (0.33, 1.26) 95% CI, *P* = 0.20] (Appendix 2).

### Secondary outcomes

The pooled analysis of emergency and elective resections showed no statistically significant difference between the two comparison groups [0.9% NABX vs. 1.1% ABX, RD: -0.00 (-0.01, 0.00) 95% CI, *P* = 0.33] and [3.2% NABX vs. 2.6% ABX, OR: 1.14 (0.54, 2.43) 95% CI, *P* = 0.73], respectively (Fig. 2).

Moreover, the two groups also showed comparable results with the following: development of complicated diverticulitis [2.6% NABX vs. 2.9% ABX, OR: 0.90 (0.56, 1.43) 95% CI, *P* = 0.65], Diverticular abscess [0.7% NABX vs. 0.3 ABX, OR 2.04 (0.47, 8.77) 95% CI, *P* = 0.34], perforation [0.9% NABX vs. 0.8% ABX, OR 1.15 (0.29, 3.35) 95% CI, *P* = 0.80], fistula [0.5% NABX vs. 0.3% ABX, OR 1.05 (0.29, 3.75) 95% CI, *P* = 0.94], re-admissions [13.6% NABX vs. 11% ABX group, OR: 1.12 (0.71, 1.77) 95% CI, *P* = 0.65] and 30-day mortality [2.6% NABX vs. 3.0% ABX, OR: 0.97 (0.57, 1.64) 95% CI, *P* = 0.91] (Fig. 2).

Length of hospital stay was reported in 10 studies (*n* = 3,430 patients). The NABX group revealed a significantly shorter length of stay compared with the ABX group [3.2 ± 2.2 days in the NABX group vs. 4.1 ± 3.2 days in the ABX group, MD: -0.68 (-1.14, -0.22) 95%, *P* = 0.004] (Fig. 2).

### B- Outpatient versus inpatient management

Our search yielded twelve studies [23, 30–40] comparing OP vs. IP management of patients diagnosed with Hinchey 1a diverticulitis (PRISMA flow chart - Fig. 1). A total of 2,286 patients were divided into the OP group (*n* = 1,021) and the IP group (*n* = 1,265). A single study was a multicentric RCT [37]; the remainder were observational studies [23, 30–36, 38–40]. Various treatment strategies were employed in the included studies. These included a conservative NABX approach [23, 36, 39], oral ABX for OP treatment and intravenous ABX for IP’s [30–33, 37, 38, 40], and finally intravenous ABX for all patients [34, 35].

Baseline characteristics of the included studies are summarised in Table 2.

**Table 2:** Baseline characteristics of included studies comparing outpatient versus inpatient treatment for patients with uncomplicated diverticulitis

**Study**	**Country**	**Study Type**	**Number** **of patients**	**Inclusion and Exclusion criteria and treatment approach**	**Hinchey stage and follow-up duration (months)** **mean ± SD/** **median(range)**
Alonso et al. [30]	Spain	Prospective Cohort	OP: 70 IP: 26	**Inclusion criteria**: uncomplicated AD with the following finding on CT scan: colonic wall thickening and/or soft tissue stranding of the pericolic fat. **Exclusion criteria**: inability to tolerate oral intake, comorbidity (diabetes mellitus, heart failure, renal insufficiency, chronic obstructive pulmonary disease) and lack of adequate family or social support. **Treatment approach**: oral ABX (OP group) and IV ABX (IP group).	**Uncomplicated AD** 39 ± 23 months
Park et al. [31]	Korea	Prospective Cohort	OP: 40 IP: 63	**Inclusion criteria**: first attack of AD, inflamed diverticulum, phlegmon formation, and < 3 cm abscess formation on CT. **Treatment approach**: oral ABX (OP group) and IV ABX (IP group).	**Uncomplicated AD** 21 months (4–40)
Lorente et al. [32]	Spain	Retrospective Cohort	OP: 90 IP: 46	**Inclusion criteria**: uncomplicated AD (CT scan: presence of diverticula with colon wall thickening (> 4 mm) or peri-colonic fat stranding), tolerance to oral intake, absence of comorbidities and adequate family or social support. **Treatment approach**: oral ABX (OP group) and IV ABX (IP group).	**Uncomplicated AD** 17 ± 5 months
Moya et al. [33]	Spain	Prospective Cohort	OP: 32 IP: 44	**Inclusion criteria**: age < 90 years, grades Ia/Ib of Ambrosetti’s AD on CT, immunocompetent, tolerating oral feeding, no severe sepsis, social support. **Exclusion criteria**: patients with complicated AD. **Treatment approach**: oral ABX (OP group) and IV ABX (IP group).	**Ambrosetti’s grades** Ia & Ib 6 months
Rueda et al. [34]	Spain	Retrospective Cohort	OP: 38 IP: 18	**Inclusion criteria**: <80 years of age, clinical signs suggesting the existence of AD and absence of clinical signs of complications such as peritonitis, vomiting, or severe abdominal distention, CT indicating Hinchey I-II, and social support. **Treatment approach**: IV ABX both OP and IP groups.	**Hinchey** I and II
Rodriguez-Cerrillo et al. [35]	Spain	Prospective Cohort	OP: 34 IP: 19	**Inclusion criteria**: patients with uncomplicated AD on CT. **Exclusion criteria**: patients with complicated diverticulitis, β-lactam allergy or who required admission to the hospital for other pathology. **Treatment approach**: IV ABX both OP and IP groups.	**Uncomplicated AD**
Ünlü et al. [36]	Netherlands	Retrospective Cohort	OP: 118 IP: 194	**Exclusion criteria**: recurrent diverticulitis, complicated diverticulitis (fistula, stenosis, Hinchey 2, 3 and 4), right-sided diverticulitis, no follow-up. **Treatment approach**: 5.9% (OP group) received ABX and 19.1% (IP group).	**Hinchey Stage** 1a 48 months
DIVER trial (Biondo et al. [37])	Spain	Multicentric RCT	OP: 66 IP: 66	**Inclusion criteria**: patients > 18 years of age with uncomplicated AD (Hinchey stage 1a) can tolerate oral intake and social support. **Exclusion criteria**: complicated AD (Hinchey stage > 1a), pregnancy or breastfeeding; on antibiotic; colorectal cancer suspicion at CT, unstable comorbid conditions; immunosuppression, intolerance to oral intake and vomiting. **Treatment approach**: oral ABX (OP group) and IV ABX (IP group).	**Hinchey stage** 1a 2 months
Estrada Ferrer et al. [23]	Spain	Prospective Cohort	OP: 36 IP: 9	**Inclusion criteria**: age 18–80, no AD episode in the last 3 months, mild AD on CT, immunocompetence (no corticosteroid therapy), no significant comorbidities (diabetes mellitus, renal insufficiency, morbid obesity), good oral tolerance, and good symptom control by oral medication. **Treatment approach**: NABX in either group (IP and OP).	**Mild AD (mNeff 0)** 6 months (3–12)
Joliat et al. [38]	Switzerland	Retrospective Cohort	OP: 171 IP: 369	**Inclusion criteria**: >18 years old and CT-based diagnosis of uncomplicated AD **Exclusion criteria**: patients requiring immediate percutaneous drainage or surgery, complicated diverticulitis (perforation, pneumoperitoneum, presence of fistula), intra-abdominal or pericolic abscess, bleeding, or stenosis. **Treatment approach**: oral ABX (OP group) and IV ABX (IP group).	**Uncomplicated AD** OP: 46.5 months IP: 59.5 months
Bolkenstein et al. [39]	Netherlands	Retrospective Cohort	OP: 264 IP: 301	**Inclusion criteria**: adult ≥ 18 years of age presenting with a first episode of uncomplicated AD on CT (Hinchey stage 1a) **Exclusion criteria**: immunocompromised with signs of sepsis or received antibiotics within 24 h after or 2 weeks before presentation. **Treatment approach**: NABX in either group (IP and OP).	**Hinchey Stage** 1a 1 month
Teke et al. [40]	Turkey	Retrospective Cohort	OP: 62 IP: 110	**Inclusion criteria**: uncomplicated AD (modified Hinchey 1a) on CT scan. **Exclusion criteria**: patients under 18 years old and complicated diverticulitis. **Treatment approach**: oral ABX (OP group) and IV ABX (IP group).	**Hinchey Stage** 1a 1 month

* OP* out-patient group, *IP* in-patient group, *AD* acute diverticulitis, *CT* computed tomography, *ABX* antibiotics, *SD* standard deviation, *RCT* randomised controlled trial, *IV* intravenous

### Primary outcome

**Fig. 3:**
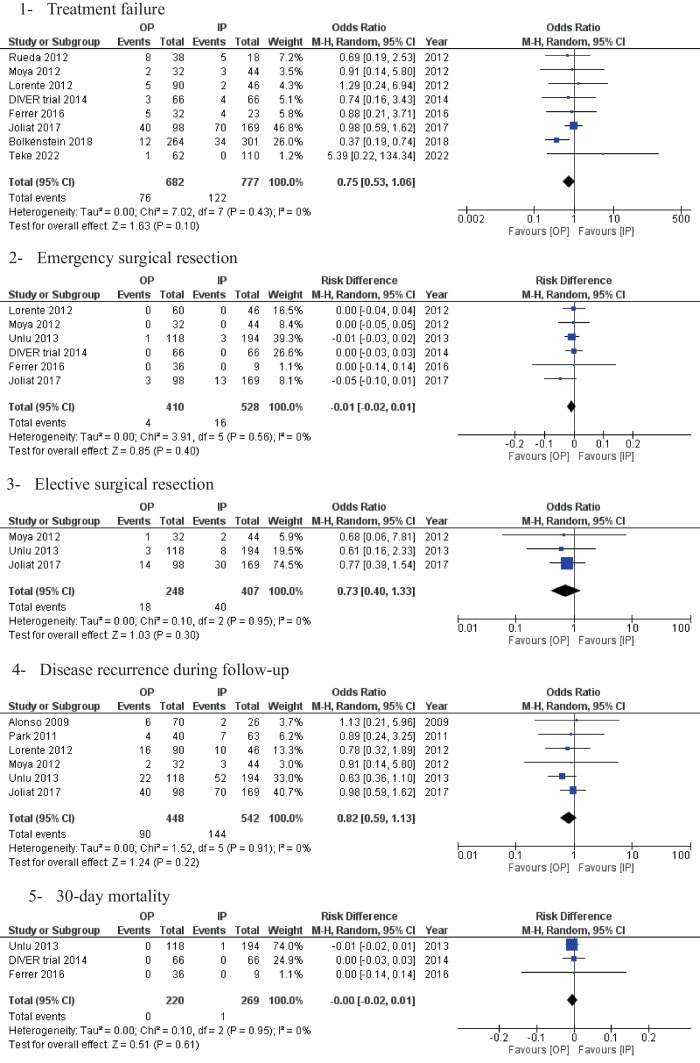
Forest plots of the measured outcomes compared between the outpatient group (OP) and the inpatient group (IP)

Treatment failure was reported in eight studies with an overall rate of 13.5% (Fig. 3). This was comparable in patients treated either in the OP or IP setting [11.1% OP vs. 15.7% IP, OR: 0.75 (0.53, 1.01) 95% CI, *P* = 0.10]. Cochran’s Q test level of heterogeneity was low between the included studies [I^2^ = 0%, *P* = 0.43].

### Secondary outcomes

Emergency surgical resection, elective resection, and recurrence rates were reported to be lower in the OP group compared to IP (non-statistically significant difference) [0.97% OP vs. 3.0% IP, RD: -0.01 (-0.02, 0.01) 95% CI, *P* = 0.40], [7.2% OP vs. 9.8% IP, OR: 0.73 (0.40, 1.33) 95% CI, *P* = 0.30], and [20% OP vs. 26.5% IP, OR: 0.82 (0.59, 1.13) 95% CI, *P* = 0.22], respectively (Fig. 3).

Moreover, the mortality rate during the follow-up period reported in three studies was also similar between the two groups [0% OP vs. 0.4% IP, RD: -0.00 (-0.02, 0.01) 95% CI, *P* = 0.61] (Fig. 3).

### Cost difference

The mean financial cost of IP vs. OP treatment of patients presenting with stage 1a acute diverticulitis was reported in four studies [31, 33, 35, 37] and was significantly lower in the latter group (Table 3).

**Table 3:** Comparison of the mean cost of outpatient vs. inpatient treatment (per patient per episode of diverticulitis)

**Study**	**Outpatient treatment** **(€ - EUR)**	**Inpatient treatment** **(€ - EUR)**	***P*-value**
Park et al. [31]	1164 ± 128	1789 ± 152	0.001
Moya et al. [33]	347.31	1945.26	< 0.05
DIVER trial (Biondo et al. [37])	547.05	1671.75	NA
Rodriguez-Cerrillo et al. [35]	The cost of each patient treated at home was 1368 EUR cheaper than those treated in the hospital.		

* NA* not available

### Sensitivity analysis

The direction of the pooled effect size remained unchanged when RR or RD was calculated for dichotomous variables. Furthermore, the leave-one-out analysis has not demonstrated important discrepancies with the original analysis.

### Risk of bias assessment

**Fig. 4:**
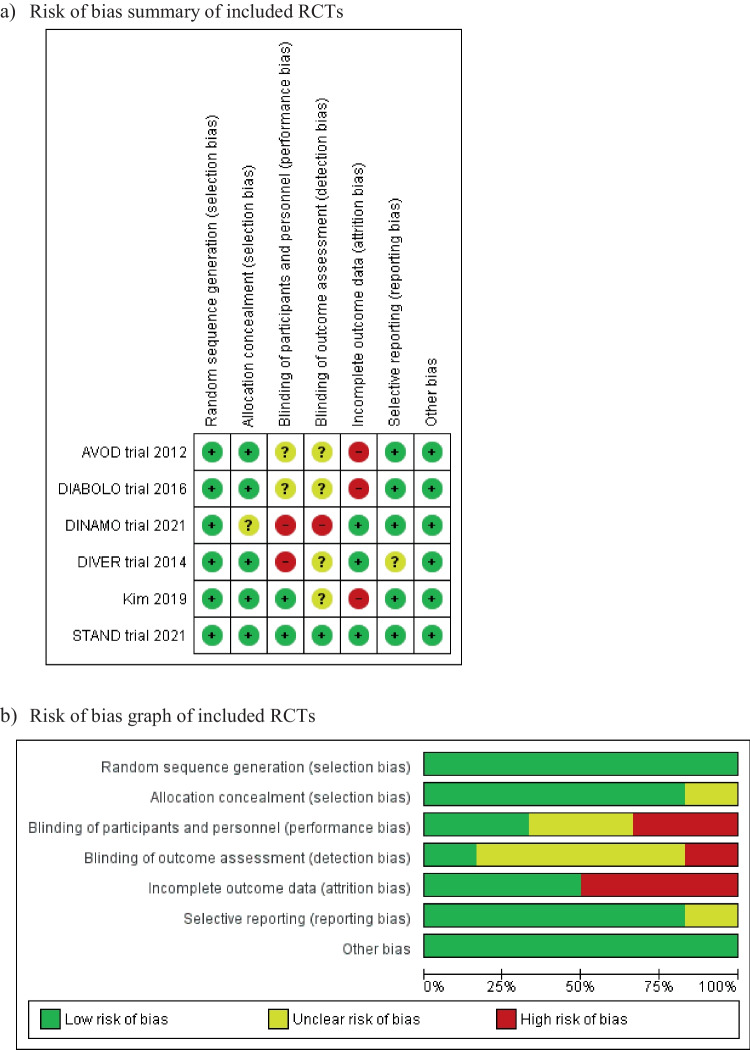
Risk of bias assessment of included randomised controlled trials (RCTs)

The included six RCTs reported random sequence generation, while allocation concealment was reported in five studies [17, 18, 21, 22, 24, 25, 27]. Blinding of participants and personnel was attempted in two studies [24, 27], whereas blinding of outcome assessor was attempted in only one study [27]. The risk of detection and performance bias remains unclear or high in the rest of the studies. Additionally, three studies were considered to have a high risk of attrition bias [17, 18, 21, 22, 24]. An overview of the risk of bias is shown in Fig. 4.

 Risk of bias assessment for the observational studies is shown in Table 4.

**Table 4:** Risk of bias assessment for observational studies using the Newcastle-Ottawa Scale

**Study**	**Representativeness of the exposed cohort**	**Selection of the non-exposed cohort**	**Ascertainment of exposure**	**Demonstration that outcome of interest was not present at start of the study**	**Comparability of cohorts based on the design or analysis controlled for confounders**	**Assessment of outcome**	**Was follow-up long enough for outcomes to occur**	**Adequacy of follow-up of cohorts**	**Total**
Hjern et al. [16]	*	*	*	*		*	*	*	7
de Korte et al. [19]	*	*	*	*	*	*	*	*	8
Brochmann et al. [20]	*	*	*	*		*	*	*	7
Estrada Ferrer et al. [23]	*	*	*	*		*		*	7
Lee et al. [26]	*	*	*	*	**	*	*	*	9
Azhar et al. [28]	*	*	*	*	*	*	*	*	8
Serrano Gonzalez et al. [29]	*	*	*	*	*	*	*	*	8
Alonso et al. [30]	*	*	*	*		*	*	*	7
Park et al. [31]									
Moya et al. [33]	*	*	*	*	*	*	*	*	8
Rueda et al. [34]	*	*	*	*		*		*	7
Lorente et al. [32]	*	*	*	*	*	*	*	*	8
Rodriguez-Cerrillo et al. [35]	*	*	*	*	*	*	*	*	8
Ünlü et al. [36]	*	*	*	*		*	*	*	7
Joliat et al. [38]	*	*	*	*		*	*	*	7
Bolkenstein et al. [39]	*	*	*	*		*	*	*	7
Teke et al. [40]	*	*	*	*	*	*	*	*	8

## Discussion

Colonic diverticulosis is common in developed countries, and complications can range from mild attacks to perforations and peritonitis requiring emergency surgery. We performed a systematic review and meta-analysis comparing the need for antibacterial therapy and IP vs. OP management in patients presenting with acute uncomplicated diverticulitis (Hinchey stage 1a).

For the former, twelve studies [16–29] with a total of 3,875 patients divided into a NABX group (*n* = 2,008) and ABX group (*n* = 1,867) were included. For management in the IP vs. OP setting, twelve studies [23, 30–40] with 2,286 patients (OP, *n* = 1,021; IP, *n* = 1,265) were included.

The resulting analysis showed a combined significantly lower risk of disease recurrence and shorter hospital stay in patients treated without ABX. Developing complicated grades of diverticulitis, hospital re-admissions, need for emergency and/or elective surgical intervention, and 30-day mortality rates were similar between the two treatment groups (ABX vs. NABX).

In addition, there was no difference between the two groups when managed as an OP in comparison with IP treatment for parameters including disease recurrence, treatment failure and mortality rates. Perhaps unsurprisingly, OP management was significantly cheaper. Our findings are in agreement with previous literature [41]. 

Several systematic reviews have reported outcomes following the omission of ABX in patients with acute uncomplicated diverticulitis [41–45]. Disease recurrence rate has previously been reported as being similar between patients treated with ABX and the NABX group [41–45], which is contradictory to our results. This could be explained by our larger sample size and the inclusion of more studies. Moreover, our meta-analysis analysed subgroups, including patients with right-sided diverticulitis and those presenting with a first episode. The most recent study by Poh et al. [45] showed similar results to the present review for outcomes, including diverticulitis complications, mortality rate, and emergency surgical intervention.

Evidence from trial and observational data suggests that routine ABX use is unnecessary. The open-label, randomised, multi-centre DINAMO study [25] demonstrated the non-inferiority of NABX treatment for hospital re-attendance, pain control, development of complications, and the need for emergency surgery. Another double-blind, placebo-controlled, multicentre RCT [27] in patients with Hinchey 1a also demonstrated non-inferiority of this approach.

The DIABLO study [21, 22] comparing ABXs with symptomatic treatment in adults with a first episode of acute uncomplicated diverticulitis reported no difference in time to recovery, with a shorter length of hospital stay in the NABX group. However, reattendance to hospital emergency departments was higher in this group. Disease recurrence and emergency surgical resection rates were identical between the two groups.

The open-label AVOD trial [17, 18] comparing ABX regimens with just intravenous fluids in patients with CT-confirmed disease also found no difference in primary outcomes, including the development of complications and the need for emergency surgical intervention. Recurrence rates and length of stay were no different between the study groups. However, long-term results suggest a possible increase in recurrent attacks and the need for surgical resection in the latter group. In addition to trial data, observational studies [46] have also demonstrated the efficacy of the NABX strategy. In a cohort of 155 patients, 97.4% were treated as OPs without the need for ABX.

Published guidelines also suggest the selective rather than routine approach to the use of anti-bacterial therapy. These include the American Gastroenterological Association Institute [47] and the World Society of Emergency Surgery [1]. The latter recommends NABX use in systemically well, immune-competent patients. In line with others, the National Institute for Health and Care Excellence (NICE) guidelines [10] also suggest adopting a NABX prescribing strategy in systemically well patients with acute diverticulitis and instead offering symptomatic treatment and a period of observation.

Cost analysis comparing oral ABXs in the community was associated with a cost saving of approximately £1100/patient compared with hospital admission and administration of intravenous ABX [37]. Based on these findings, an NABX approach in an OP setting would presumably lead to even greater cost savings in depleted healthcare systems.

Despite the presence of mainly low-quality and sparse evidence, uncomplicated acute diverticulitis has routinely been treated with antibacterial therapy. NABX management for uncomplicated disease was first described by Hjern et al. [16] and appeared to be safe with no increase in the likelihood of adverse events. This thinking was further challenged as acute diverticulitis was thought of as an inflammatory disorder rather than an infectious condition, further questioning the rationale for the use of ABXs.

Extensive, unwarranted ABX use has several drawbacks, including financial costs, risk of adverse events, and the development of opportunistic severe infections (clostridium difficile) [48]. Additionally, the overuse of ABXs is a real concern for increasing antimicrobial resistance (AMR) and reducing the clinical efficacy of these drugs.

This meta-analysis is not without its limitations, which need to be considered when interpreting our findings. The main limitation of this review is that most of the included studies are observational and inherently carry a high risk of selection bias. To overcome this risk, we performed subgroup analysis for RCTs alone for the primary outcome (disease recurrence rate). This showed no significant difference between the two groups (ABX vs. NABX) compared with the significant difference seen when all studies are included. This difference could be due to the allocation of elderly/frail and unwell patients with clinical risk for recurrence to the ABX group. Some of the reported outcomes showed high heterogeneity due to the various methodologies employed by individual studies. Moreover, data regarding type, route, and duration of ABX use was understandably varied between the studies as the antimicrobial guidelines differ between centres and geographical locations in which these studies were conducted.

The duration of follow-up was inconsistent amongst the included studies and insufficient for long-term outcomes to be assessed in a robust and vigorous manner. Despite the aforementioned limitations and the fact that several meta-analyses have been published investigating the role of NABXs in treating uncomplicated acute diverticulitis, we believe this review is unique as it is the first to demonstrate ABX treatment increases the risk of disease recurrence. Additionally, by including recently published studies, this review provides an update to the available evidence supporting a NABX approach and the OP management for patients presenting with uncomplicated acute diverticulitis.

## Conclusions

Observation-only treatment is feasible and safe in selected clinically stable patients with uncomplicated acute diverticulitis (Hinchey 1a classification). It may decrease the length of hospital stay and the risk of disease recurrence. Moreover, the OP approach can be considered in carefully selected patients. Future rationally designed, well-powered, randomised, placebo-controlled trials are needed to understand the outcomes of the NABX approach used in an OP setting in managing patients with uncomplicated acute diverticulitis.

## Supplementary Information

Below is the link to the electronic supplementary material.

Supplementary file1 (DOCX 14.2kb)

Supplementary file2 (DOCX 72.2 kb)

## Data Availability

No datasets were generated or analysed during the current study.
