# Supplementary material for: Systematic review and meta-analysis of the management of acute uncomplicated diverticulitis: time to change traditional practice
Source: Int J Colorectal Dis. 2024 Apr 5;39(1):47. doi: 10.1007/s00384-024-04618-7 (PMC10997545; doi:10.1007/s00384-024-04618-7)
Supplement: Supplementary file 1 — Supplementary file1 (DOCX 14.2kb) [file 384_2024_4618_MOESM1_ESM.docx]

**Search strategy**

1. **No antibiotics versus antibiotic treatment for uncomplicated diverticulitis.**

(("uncomplicated"[All Fields] AND ("diverticulitis"[MeSH Terms] OR "diverticulitis"[All Fields])) OR ("diverticular diseases"[MeSH Terms] OR ("diverticular"[All Fields] AND "diseases"[All Fields]) OR "diverticular diseases"[All Fields] OR ("diverticular"[All Fields] AND "disease"[All Fields]) OR "diverticular disease"[All Fields]) OR (("hinchey"[All Fields] OR "hinchey s"[All Fields]) AND ("stage"[All Fields] OR "staged"[All Fields] OR "stages"[All Fields] OR "staging"[All Fields] OR "stagings"[All Fields]) AND "1a"[All Fields]) OR ("diverticulitis"[MeSH Terms] OR "diverticulitis"[All Fields]) OR ("diverticulitis, colonic/complications"[MeSH Major Topic] OR "diverticulitis, colonic/drug therapy"[MeSH Major Topic] OR "diverticulitis, colonic/therapy"[MeSH Major Topic])) AND ("anti bacterial agents"[Pharmacological Action] OR "anti bacterial agents"[MeSH Terms] OR ("anti bacterial"[All Fields] AND "agents"[All Fields]) OR "anti bacterial agents"[All Fields] OR "antibiotic"[All Fields] OR "antibiotics"[All Fields] OR "antibiotic s"[All Fields] OR "antibiotical"[All Fields] OR ("observability"[All Fields] OR "observable"[All Fields] OR "observables"[All Fields] OR "observation"[MeSH Terms] OR "observation"[All Fields] OR "observe"[All Fields] OR "observed"[All Fields] OR "observer"[All Fields] OR "observer s"[All Fields] OR "observers"[All Fields] OR "observes"[All Fields] OR "observing"[All Fields] OR "watchful waiting"[MeSH Terms] OR ("watchful"[All Fields] AND "waiting"[All Fields]) OR "watchful waiting"[All Fields] OR "observations"[All Fields]) OR ("anti bacterial agents"[Pharmacological Action] OR "anti bacterial agents"[MeSH Terms] OR ("anti bacterial"[All Fields] AND "agents"[All Fields]) OR "anti bacterial agents"[All Fields] OR "antibiotic"[All Fields] OR "antibiotics"[All Fields] OR "antibiotic s"[All Fields] OR "antibiotical"[All Fields]) OR "non-antibiotics"[All Fields])

1. **Outpatient versus inpatient treatment of uncomplicated diverticulitis**

(("uncomplicated"[All Fields] AND ("diverticulitis"[MeSH Terms] OR "diverticulitis"[All Fields])) OR ("diverticular diseases"[MeSH Terms] OR ("diverticular"[All Fields] AND "diseases"[All Fields]) OR "diverticular diseases"[All Fields] OR ("diverticular"[All Fields] AND "disease"[All Fields]) OR "diverticular disease"[All Fields]) OR (("hinchey"[All Fields] OR "hinchey s"[All Fields]) AND ("stage"[All Fields] OR "staged"[All Fields] OR "stages"[All Fields] OR "staging"[All Fields] OR "stagings"[All Fields]) AND "1a"[All Fields]) OR ("diverticulitis"[MeSH Terms] OR "diverticulitis"[All Fields]) OR ("diverticulitis, colonic/complications"[MeSH Major Topic] OR "diverticulitis, colonic/drug therapy"[MeSH Major Topic] OR "diverticulitis, colonic/therapy"[MeSH Major Topic])) AND ("outpatient s"[All Fields] OR "outpatients"[MeSH Terms] OR "outpatients"[All Fields] OR "outpatient"[All Fields] OR ("ambulatories"[All Fields] OR "ambulatory"[All Fields]))
