# Supplementary material for: Systematic review and meta-analysis of the management of acute uncomplicated diverticulitis: time to change traditional practice
Source: Int J Colorectal Dis. 2024 Apr 5;39(1):47. doi: 10.1007/s00384-024-04618-7 (PMC10997545; doi:10.1007/s00384-024-04618-7)
Supplement: Supplementary file 2 — Supplementary file2 (DOCX 72.2 kb) [file 384_2024_4618_MOESM2_ESM.docx]

1. Subgroup analysis of RCTs and observational studies for disease recurrence during follow-up


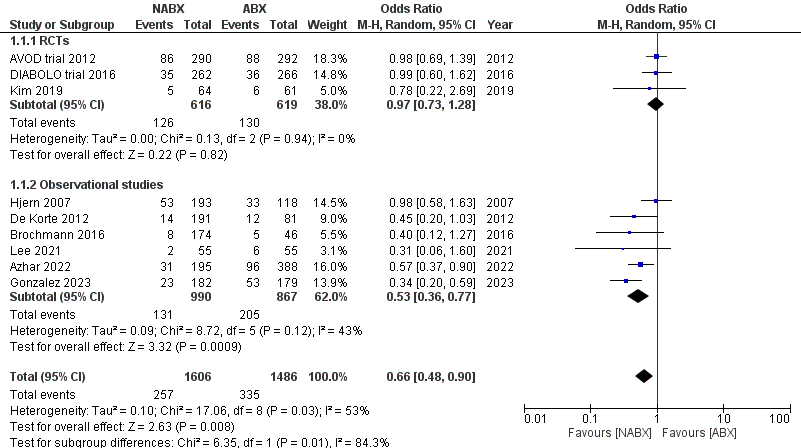


1. Subgroup analysis of studies that included exclusively right-side AD for disease recurrence during follow-up


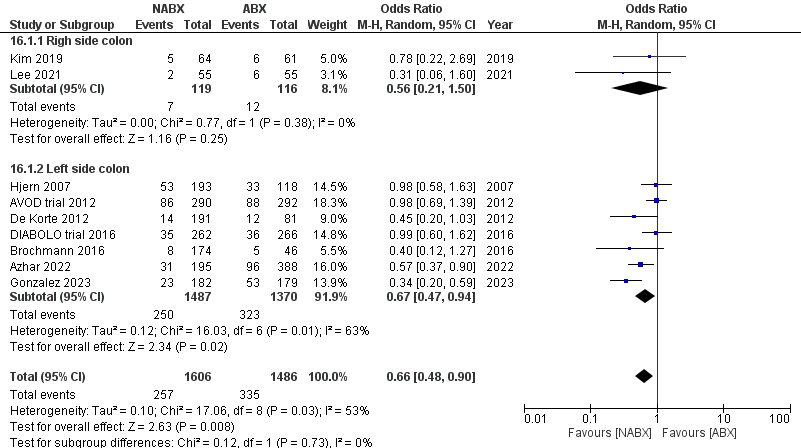


1. Subgroup analysis of studies that included patients with first episode of AD exclusively for disease recurrence during follow-up


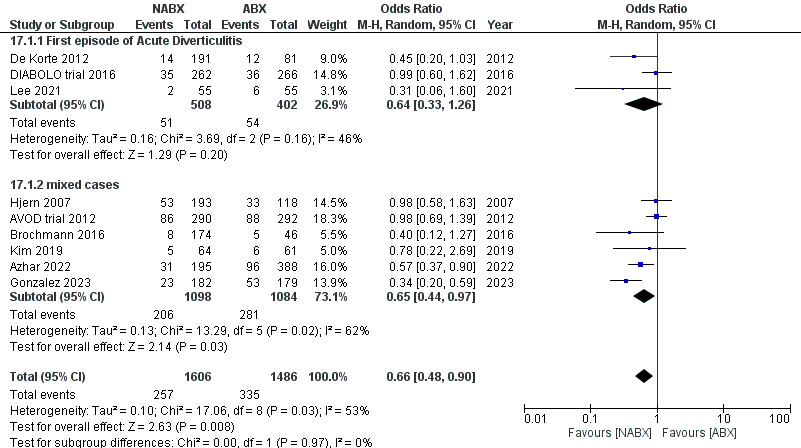


Appendix 2: Forest plots of the subgroups for recurrence compared between the no-antibiotic group (NABX) and the antibiotic group (ABX).
